# Cancer prevalence in the city of Naples: Contribution of the GP database analyses to the cancer registries network

**DOI:** 10.3892/mco.2013.101

**Published:** 2013-04-04

**Authors:** CLAUDIA PIZZI, GRAZIA ARPINO, GIUSEPPE ACAMPORA, NADIA AIELLO, AUGUSTO DE ROSA, IMMACOLATA DIAFERIA, ALESSANDRO DI NUNZIO, GIUSEPPE FRAGNA, AMEDEO FRANCO, MARIA RUSSO, FULVIA SANSONE, CARMELA SCARPATI, ANTONIO SPINUSO, GIOVANNI ARPINO, AMALIA LUCE, GIUSEPPINA TOMMASIELLI, MICHELE CARAGLIA, SABINO DE PLACIDO

**Affiliations:** 1 Consorzio Nazionale delle Cooperative Mediche - Italian Society of General Medicine (SIMG), ‘Federico II’ University, 80131 Naples;; 2 Department of Clinical Medicine and Surgery, Faculty of Medicine, ‘Federico II’ University, 80131 Naples;; 3 Department of Biochemistry, Biophysics and General Pathology, Second University of Naples, 80138 Naples, Italy

**Keywords:** cancer prevalence, Naples, general practitioners, tumour registries

## Abstract

The Italian cancer registries network has not been sufficiently developed in the Southern regions. General practitioners (GPs) are knowledgeable about the prevalence, incidence and mortality for different types of cancer in their patient populations. The aim of this pilot study was to verify the feasibility and reliability of the characterization of cancer populations using GP databases in order to evaluate the impact of cancer in the general population of Naples. The characteristics of the cases studied have been collected by interview or electronic health record and recorded on paper or magnetic supports, appropriately conforming to the current privacy law. Databases are centralized, stored and codified on electronic data-sheets and periodically elaborated by the ‘Consorzio Nazionale delle Cooperative Mediche’ and ‘Federico II’ University. The present study was initiated on September 15, 2004. The analysed geographical area included the suburbs of ‘Stella’ and ‘San Carlo all’Arena’, situated in the historical center of Naples and corresponding to Health Care District 29 of the local health service. The analysis included 16,927 men and women (age range, 6–97 years) from the outpatient offices of 12 GPs who agreed to participate in the study. Results showed that the analysed population represents 16.3% of the general population residing in the area under study. We identified 342 (2%) patients with cancer, 143 (0.8%) of whom were men and 199 (1.2%) women (M/F ratio of 0.7). Of the 342 patients, 10 (5 men and 5 women) had a double cancer; thus, a total of 352 malignancies was characterized. Cancer prevalence was 2,020/100,000 inhabitants. This estimate is lower compared to the national prevalence (2,683/100,000 inhabitants) but higher compared to that in other southern Italian areas. Results, stratified by International Classification of Disease, ninth revision (ICD-IX), based on factors including gender and age, demonstrated that breast cancer, urogenital tumours and colorectal cancer are the most frequently occurring types of cancer identified among the inhabitants of Naples. Cancer prevalence in the historical center of Naples is in concordance with national estimates and projections and National Cancer Registries may be easily and accurately supported by GP medical databases.

## Introduction

The marked increase in the incidence of cancer worldwide requires public health actions by governments and health officers to control this trend and implement preventive measures (1–3). At present, however, despite scientific efforts to improve the outcome of cancer patients, an increased cancer incidence leads to an increased likelihood of death, particularly in certain tumour subtypes and in certain geographical areas (4–6). It is important to have systematically available population-based survival data for cancer patients in order to aid health planners in allocating resources (1–6).

Data reported by the WHO for the year 2000 clearly demonstrated that cancer had led to 12% of ∼56 million deaths worldwide. More importantly, of the 5.3 million men and 4.7 million women that developed a cancer, over half of the individuals (6.2 million), succumbed to this disease (1–3). Surveillance of the impact of cancer on the general population by cancer registries is the most powerful approach to estimate the magnitude of the problem and plan appropriate strategies to prevent the onset of the disease or, where not possible, to improve measures for early detection. Moreover, information concerning the incidence, prevalence, mortality and survival allows us to assess the outcomes of screening and diagnostic procedures and the effect of preventive and therapeutic interventions (4–9).

In 2006, individuals suffering from cancer (i.e., the prevalence) in Italy numbered to 2,244,000 (4% of the Italian population), with a proportion of 4–5% in the Northern-Central regions and 2–3% in the Southern regions, where the mortality rate is more elevated. Female outnumbered male survivors (56 vs. 44%). The Italian estimates are similar to those of Northern Europe, but at least 15% lower compared to those in the United States (1–6,10).

In Italy, the cancer registries network is comparable to the surveillance systems present in other industrialized countries, although in the Southern regions of the country it is inadequately represented (7–13). The lack or malfunctioning of cancer registries in most of the Southern Italian regions explains the wide variability in the information available on cancer prevalence and incidence in different areas of the Italian peninsula. The bulk of epidemiological data on the Southern Italian population of cancer patients originates from mortality and hospitalization records (13–17). All the evidence available, however, have limitations due to study design, confounder elements and quality of exposure data (18–19). However, the deficiency of a comprehensive database collecting the most relevant clinical and biological tumour characteristics causes a false or confounding perception of the problem in these areas (18,19).

Data on cancer prevalence, incidence and mortality in Campania, one of the most heavily populated Southern Italian regions, are sparse and fragmentary and focus predominantly on detailed geographical areas near landfills or single waste sites (14–23). Campania is also the region with the largest economic deficit due to health expenses. Obtaining information concerning the impact of cancer incidence and prevalence in this area may be critical to appropriately employ human and economic resources, given the high costs of treatment and diagnostic procedures. An ongoing update database recording patient clinical and disease characteristics as well as treatment outcomes may therefore be of great benefit in the development of a coherent public health program.

In Italy, the National Health Services provide a general practitioner (GP) to each citizen. The GP is familiar with the risk factors, co-morbidities and diseases, including cancer, affecting their particular patient population. Regarding patients with cancer, data on cancer characteristics, the diagnostic and therapeutic procedures performed on the patient, the possible reasons for the selection of one treatment over another and the clinical outcome are carefully recorded in the GP’s clinical records. Therefore, the GP clinical chart databases contain all the most relevant information required to produce an effective surveillance program on the cancer patient population.

This is a pilot study evaluating the feasibility and reliability of the characterization of the oncology population of a central zone of Naples through the analyses of the medical records of GPs practicing in that area. In the present study we also evaluated the impact of cancer on the general population residing in the center of Naples by reviewing GPs’ clinical charts. Relevant clinical characteristics such as age, gender and site, based on the International Classification of Disease, ninth revision (ICD-IX), were analyzed in GP medical databases for cancer patients residing in that area and the cancer prevalence in Naples was determined.

## Patients and methods

### General

General practitioner databases (GPDs) containing health information of patients residing in the center of Naples and belonging to the local health service area ‘ASL Napoli 1 Centro’ were retrieved. The patients included in the study were assisted by a GP belonging to the GPs cooperative Group ‘MEDI.CO’. This service society has been acknowledged by the General Practitioner Regional Agreement (D.P.R. 270/2000) to be appropriate for the elaboration of population-based clinical studies. The MEDI.CO cooperative Group is part of the Consorzio Nazionale delle Cooperative Mediche and operates according to the scientific indications of SIMG (Italian Society of General Medicine).

Cancer patients were identified if one of the following criteria was present: registration of an ICD-IX revision code diagnostic for malignant cancer (ICD-IX 140-208) based on histological confirmation or on the presence of the disease code p048 malignant cancer in health records. Clinical information and disease characteristics of eligible patients were also searched in the computerized or paper-based clinical charts and data registered on preformed standardized sheets. Each GP participating in the study, identified the cancer patients in his own patient population and filed a summary diagram showing the number of patients suffering from cancer compared to the total population. Cancer patients were further classified according to the histotype and particular conditions (metastases, double or second tumour).

Data were recorded in a dedicated computerized database organized for the study using Microsoft Excel software spreadsheets, according to a standardized shared coding. Informed consent was obtained from each participant and data were assessed according to the requirements of the national privacy law. Data analyses were periodically performed in collaboration with the Department of Oncology of the University of Naples ‘Federico II’ (24) (http://annonc.oxfordjournals.org/content/16/suppl_7/vii64.full.pdf).

Primary endpoints of the study were estimation of the impact of cancer on the general population residing in the center of Naples by analyzing the prevalence and verification of the feasibility and reproducibility of tumour dynamic registries performed by GPs on their GPDs.

A secondary objective was the investigation of the distribution and predictive role of recently debated onco-genetic risk factors such as gender, age and socio-economic position in an identified cancer patient population, as in the case of the inhabitants of Campania (14–20).

### Statistical analysis

**Figure 1.**
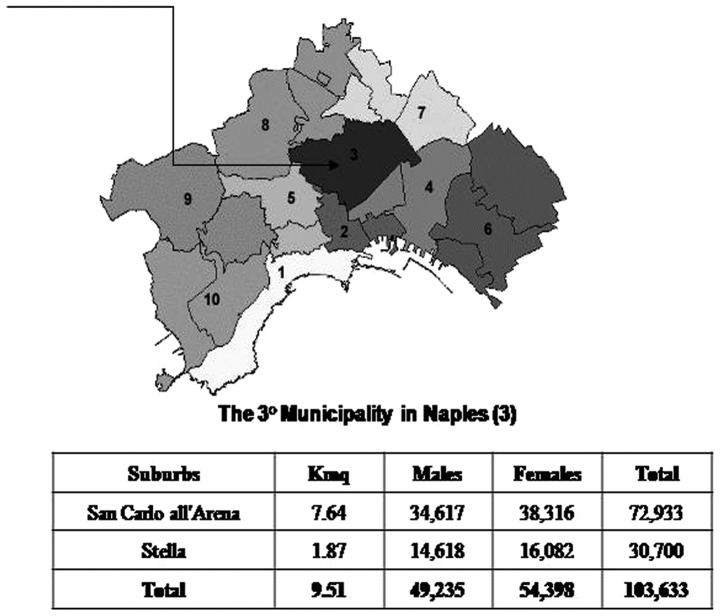
Geographical and demographic characteristics of the third Municipality of Naples (3).

The study was initiated on September, 2004 (24). The patient population examined in the study were from the ‘Stella’ and ‘San Carlo all’Arena’ geographical areas, located in the historical center of Naples and representing the largest population governed by a single municipality in Europe (3rd Municipality of Naples), with 49,235 men and 54,938 women (Fig. 1). This Municipality corresponds with the Health Care District 29 (D29; 103,633 patients) in the ‘ASL Napoli 1 Centro’ (1,004,500 units) with a M/F ratio of 91.74/100, which is lower compared to that in the rest of Italy (M/F 93.8/100; Censimento 2001, www.comune.napoli.it).

Municipalities are the smallest administrative units (8,100 in all of Italy) where demographic and mortality data are routinely available. First, we examined the cancer prevalence in the district (D29). The distribution of cancer according to patient gender and tumour histotype was then analyzed. Prevalence was defined as the number or proportion of individuals with a previous diagnosis of cancer belonging to the specific population.

Tumours were divided according to ICD-IX and were grouped as follows: gastrointestinal sites (tongue, mouth, pharynx, oesophagus and stomach) ICD-IX 141–151; colon and rectum ICD-IX 153–154; liver, biliary system and pancreas ICD-IX 155-157; larynx ICD-IX 161; lung ICD-IX 162; bone and soft tissue sarcomas ICD-IX 170-171; melanoma and skin ICD-IX 172–173; breast cancer ICD-IX 174-175; reproductive system ICD-IX 180-187; kidney and bladder ICD-IX 188–189; thyroid gland ICD-IX 193; central nervous system (CNS) ICD-IX 190–191; leukaemia and lymphomas (blood) ICD-IX 200–205 (Tables I and II).

## Results

The study was conducted on a total of 16,927 individuals (patients and healthy subjects) followed for primary care by the 12 GPs belonging to the MEDI.CO cooperative Group who agreed to participate in the study. All 12 GPs were employed in Health Care District 29. The general population age ranged from 6 to 97 years. Of note, the population represented in this study corresponds to 16.3% of the general population residing in the geographical areas involved.

Patients with cancer represented 2% (342/16,927) of the total population. Based on these data, the overall cancer prevalence was estimated to be 2,020/100,000 inhabitants. Each GP recruited in the study reported a cancer prevalence range of 1.1–3%.

**Figure 2.**
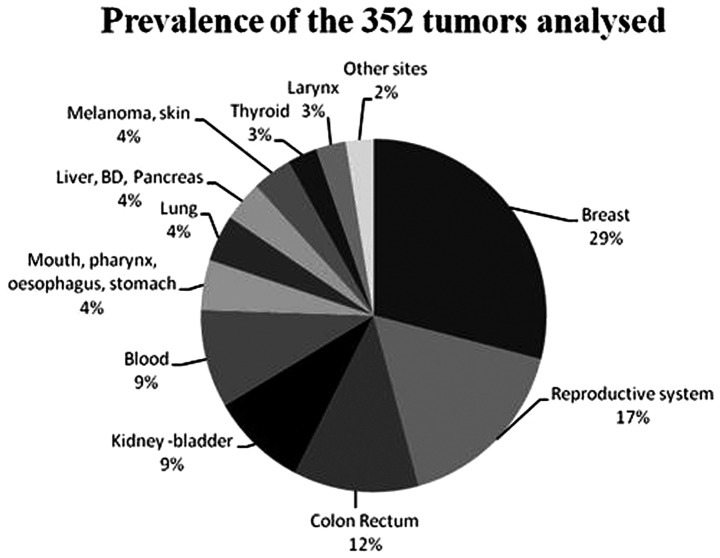
Distribution of different types of cancer by primary site in the cancer population. BD, bile duct.

We identified 342 patients with invasive cancer: their median age was 65 years and ranged from 10 (leukaemia) to 93 (epithelioma) years. The majority of the cancer cases were recorded among women (199 cases, 1.2%) compared to men (143 cases, 0.8%) (M/F ratio, 0.7). Of the 342 cancer patients, 10 patients (5 men and 5 women), were affected by more than one primary cancer; thus, for the purpose of our analyses, a total of 352 malignant cancers were considered. Fig. 2 shows the distribution of cancer by primary site in the cancer population.

Among all histotypes, breast cancer was the most frequent (29%, 102/352) among affected patients. Cancers of the reproductive system, i.e., cervix, uterus, ovary, vulva, prostate, testicle and penis, were the second most prevalent group (17%, 59/352). Among these, prostatic cancer (30/352) in men and uterine cancer (19/352) in women were the most frequent reproductive histotypes. Among gastrointestinal (GI) cancers, colorectal cancer represented 41/352 cases (12%), whereas upper GI tract histotypes were recorded in only 4% (16/352) of the cases. Of note, kidney and bladder cancer was identified in 32/352 cases, with a prevalence of 9%, identical to that of blood malignancies. All other histotypes taken together did not exceed 4% of the cases. The most frequently observed types of cancer were breast, urogenital and colorectal cancers.

Results recorded regarding different histotypes and ICD-IX were analyzed according to gender and age range of the patients. Tables I and II show the prevalence of different cancer types in males and females.

**Figure 3.**
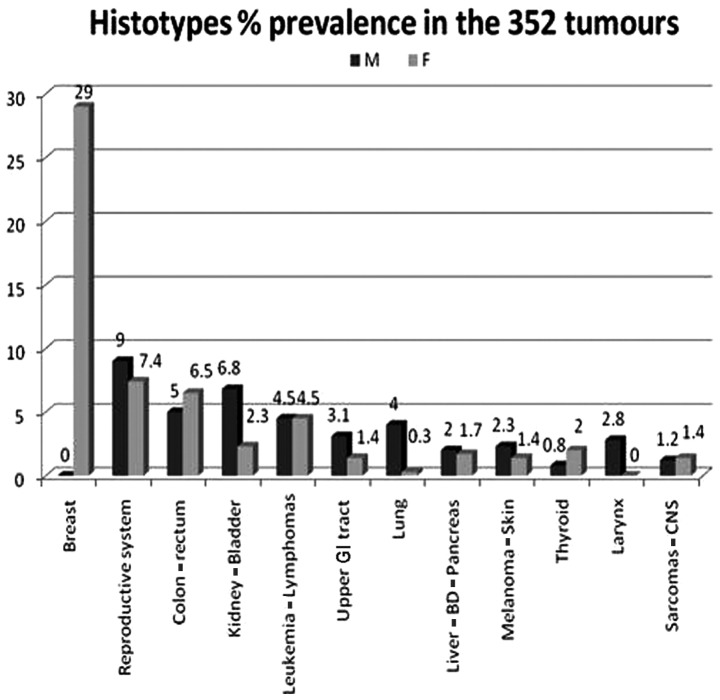
Cancer prevalence proportions in males and females. BD, bile duct; GI, gastrointestinal; CNS, central nervous system.

Among the 352 tumours, 148 cases were recorded in males and most of these were observed in the age range of 65–74 years (42.6%, 63/148). Prostate cancer was the most frequent type of tumour among males (20%, 30/148), followed by kidney and bladder tumours (16%, 24/148), which exhibited a prevalence identical with that of laryngeal and pulmonary tumours (16%, 24/148). Colorectal cancer was also identified among males (12%, 18/148). Among the 352 tumours, 204 cases were recorded in females, predominantly in the age range of 35–64 years (54.4%, 111/204). Breast cancer was the most frequent type of tumour among females (50%, 102/204), followed by colorectal (11%, 23/204) and uterine (9%, 19/204) cancers. Fig. 3 shows the percentages of prevalence distributed according to gender.

## Discussion

The almost complete lack of tumour registries in Southern Italy and, most importantly, in the Campania region, one of the most crowded and polluted areas of the Italian peninsula, makes the estimation of the annual rate of new cancer cases and cancer prevalence in this district challenging (7,10,13–18). Furthermore, the impact of legal or illegal landfills in rural and urban areas of Campania, such as neighborhoods situated around Naples or Caserta, on population health and, particularly, on the risk of cancer development, may not be accurately determined due to lack of epidemiological data in the entire region (10,14–19). However, this issue is a major health concern for the population residing in these areas and there is a pressing need for definitive answers.

Over the last few years there has been an ongoing effort, directed towards the creation of tumour registries that cover the entire Campania area. At present, however, the only available and reliable source of data on cancer incidence, prevalence and mortality in Campania remains the tumour registry of the local health service no. 4, also known as ‘Azienda Sanitaria Locale’ Napoli 4 (ASL Napoli 4), that covers only a small part of the entire Campania population (16). Data on cancer prevalence, incidence and mortality in Campania, one of the most heavily populated regions of Southern Italy, are sparse and fragmentary and focus predominantly on detailed geographical areas near landfills or single waste sites (10,14–23).

GPs in Italy are responsible for primary and secondary cancer prevention and, more importantly, are the only ones entitled to prescribe Day Hospital admission or patient hospitalization for cancer treatment, provide medical exemption certificates for cancer patients and prescribe medication for the treatment of cancer or cancer-related symptoms. Therefore, GPs keep reliable records on the number of cancer patients among their patient population, as well as on diagnostic and therapeutic methods. Several GPs practicing in the same district in Campania and, to a certain extent, in the rest of Italy, operate in groups called ‘service societies’. Several service societies have also joined together in an organization called Consorzio Nazionale delle Cooperative Mediche. This consortium currently comprises >500 GPs, assisting ∼844,297 patients in the entire region of Campania. GPs belonging to this network record patient clinical information in commonly shared software and participate in innovative research protocols or clinical courses to improve their clinical skills and patient information retrieval through electronic patient charts, in order to ensure uniformity of data entry and extraction. At present, there is an ongoing analysis on cancer prevalence and incidence in the entirety of Campania.

In our study, the MEDI.CO service society serve the suburbs of ‘Stella’ and ‘San Carlo all’Arena’, which are located in the historical center of Naples. Of note, the patient population registered with MEDI.CO GPs represents 16.3% of the general population residing in these two geographical areas. Data from clinical patient charts retrieved from GPs belonging to the MEDI.CO society are therefore representative of the cancer prevalence and incidence in the metropolitan area of Naples. Therefore, in the present study we aimed to test the reliability of MEDI.CO GP clinical databases in the assessment of cancer prevalence and histotype distribution for the population residing in the ‘Stella’ and ‘San Carlo all’Arena’ (3rd Municipality of Naples) suburbs registered with MEDI. CO practitioners.

Overall, cancer patients represented 2% (342/16,927) of the entire population considered. For each GP recruited in the study, the recorded cancer prevalence range was 1.1–3%. This is in concordance with the previously reported cancer prevalence in the Campania area according to indirect estimates from the ‘ASL Napoli 4’ tumour registry. In agreement with national data, in our dataset females appeared to be more frequently affected by cancer compared to males (10).

The most common cancer histotypes among men were prostatic, bladder, colon, lung and laryngeal cancer and among women breast, colon and uterine cancer. The prevalence of prostatic cancer was higher among older men, whereas the prevalence of bladder cancer was not significantly affected by age. Lung, colon and laryngeal cancer in our dataset was more prevalent among middle-aged men and breast cancer was the most prevalent histotype among middle-aged women, followed by uterine cancer. There were no differences according to age in the prevalence of colon cancer. The overall cancer prevalence was slightly lower compared to that in the general Italian population (2,683/100,000 inhabitants), but higher compared to the prevalence previously reported in different Southern Italian cities (7,8,10,12,13). However, histotype distribution according to gender and age range were in accordance with the expected rates estimated from data obtained from tumori.net, the most accredited source of epidemiological data for cancer disease (http://www.tumori.net/it/banca_dati/query.php) and recent epidemiological national data (7–10).

The present study demonstrated that evaluating cancer prevalence from GP patient charts is feasible, cost-effective and reliable, provided that the patient population included in the service society is representative of the total population of the area registered with the GPs clinical practice. Therefore, the width of the GP network is positively correlated with the extent of data retrieval from a specific region and the relative measures of cancer incidence and prevalence.

Currently, in Campania, epidemiological data on cancer prevalence estimated from GP patient charts is the single and most complete data source available. However, reviewing GP patient charts is not a substitute for specific cancer registries, which remain the most reliable source of epidemiological data for cancer. Data on cancer patients obtained from GPs may corroborate information collected from tumour registries and also provide further insight on patient co-morbidities, types of treatment performed and geographical distribution of hospitals and centers specializing in cancer care. Careful interpretation of data collected from GP databases is required, considering all potential biases due to the non-homogeneous distribution of GP practices among different areas of Campania and its largest cities. Further development of the GP service society network is required, in order to optimize data collection and retrieval and provide an almost complete coverage of the whole Campania region and, in the future, the entire Italian peninsula.

## Figures and Tables

**Table I.** Cancer prevalence in males and age ranges.

ICD-IX	Sites	Age (years)		Range 65–74	≥75	Total
		10–34	35–64			
141	Tongue			2		2
143–145	Mouth			1		1
146–148	Pharynx			1	1	2
150	Oesophagus				1	1
151	Stomach		2	2	1	5
153	Colon		3	9	3	15
154	Rectum			2	1	3
155	Liver			4	1	5
156	Biliary system		1			1
157	Pancreas				1	1
161	Larynx		3	6	1	10
162	Lung		5	7	2	14
170	Bone	1	1			2
171	Soft tissue				1	1
172	Melanoma		3	3	1	7
173	Skin			1		1
185	Prostate		2	15	13	30
186	Testicle	1	1			2
187	Penis		1			1
188	Bladder		7	4	6	17
189	Kidney			4	3	7
191	CNS		1			1
193	Thyroid		3			3
201	HL	2	1			3
200,202	NHL		4		1	5
202–205	Leukaemia	2	2	2	2	8

	All tumours (%)	6 (4)	40 (27)	63 (42.6)	39 (26.4)	148

ICD-IX, International Classification of Disease, ninth revision; CNS, central nervous system; HL, Hodgkin lymphoma; NHL, non-Hodgkin lymphoma.

**Table II.** Cancer prevalence in females and age ranges.

ICD-IX	Sites	Age (years)		Range 65–74	≥75	Total
		10–34	35–64			
141	Tongue		1			1
143–145	Mouth		2			2
146–148	Pharynx		1			1
150	Oesophagus				1	1
151	Stomach					0
153	Colon		7	7	7	21
154	Rectum			2		2
155	Liver		1	1		2
156	Biliary system			1		1
157	Pancreas			3		3
161	Larynx					0
162	Lung		1			1
170	Bone	1				1
171	Soft tissue				1	1
172	Melanoma		2			2
173	Skin			1	2	3
174	Breast	2	63	23	14	102
180	Cervix		4			4
182	Uterus		11	6	2	19
183	Ovary	1	1			2
184	Vulva		1			1
188	Bladder		1	1		2
189	Kidney	1	2	1	2	6
191	CNS		1	1	1	3
193	Thyroid	1	5	1		7
201	HL	1				1
200,202	NHL		4	3		7
203	Myeloma			1		1
202–205	Leukaemia		3		4	7

	All tumours (%)	7 (3.4)	111 (54.4)	52 (25.5)	34 (16.7)	204

ICD-IX, International Classification of Disease, ninth revision; CNS, central nervous system; HL, Hodgkin lymphoma; NHL, non-Hodgkin lymphoma.
